# Oxidative Stability of Vegetal Oil-Based Lubricants

**DOI:** 10.1021/acssuschemeng.0c06988

**Published:** 2021-01-19

**Authors:** Clarissa Murru, Rosana Badía-Laíño, Marta E. Díaz-García

**Affiliations:** † Department of Physical and Analytical Chemistry, Faculty of Chemistry, University of Oviedo, Julián Clavería 8, Oviedo 33006, Asturias, Spain

**Keywords:** Vegetal-based lubricants, Oxidative stability, Oxidation mechanisms, Antioxidant additives, Chemical modifications

## Abstract

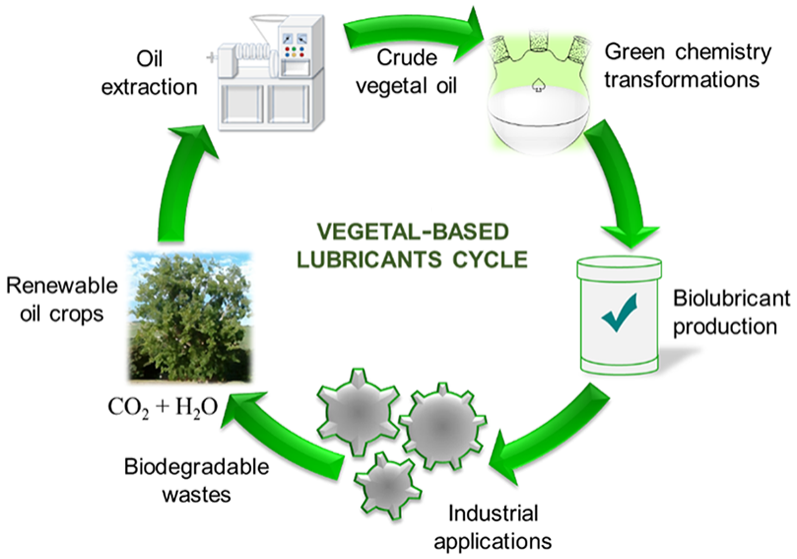

Lipids are widely distributed in nature and are one of the most important components of natural foods, synthetic compounds, and emulsions. To date, there is a strong social demand in the industrial sector for the use of sustainable products with a minimal environmental impact. Depending on their origin and composition, lipids can be employed as a plausible alternative as biodegradable lubricants in order to reduce the use of conventional mineral oil lubricants and mitigate their environmental impact. This perspective provides an overview of the advantages and constrains of vegetal oils under different lubrication regimes and the tribochemical reactions that can take place. Also, the different factors and pathways that influence their oxidation, the key role of moisture, and the changes of physical properties under pressure and temperature are reviewed. Special emphasis is devoted to the oxidation instability of fatty acids and vegetal oils and the physical and chemical approaches to improve oxidative and thermal stability are described in detail.

## Abstract 2

Vegetal oils, through sustainable chemical modifications, are a biodegradable and renewable source to replace lubricant mineral oils.

## Introduction

Lipids are a major component of food, key functional constituents of cells in biological systems, and a primary source of fuel for living organisms. Commonly, lipids are defined as substances that are insoluble in water but soluble in organic solvents.^1^ Due to the complexity and heterogeneity of lipids, an accurate definition is difficult, and different classification schemes have been used. So, lipids can be categorized based on their chemical backbone structure (simple or complex), their physical properties at room temperature (liquids as oils and solid as fats), their polarity (polar and neutral lipids), or their essentiality for humans (essential and nonessential). In 2005, Fahy et al.^2^ proposed a novel definition and a comprehensive system of classification: lipids are small hydrophobic or amphiphilic molecules that may originate entirely or in part through condensations of thioesters and/or isoprene units, a definition that enables cataloguing lipids into eight categories (fatty acids, glycerolipids, glycerophospholipids, sphingolipids, sterol lipids, prenol lipids, saccharolipids, and polyketides), each containing distinct classes and subclasses of molecules. The proposed lipid classification is compatible with other existing lipid databases and expandable to new categories in the future.^2^

Fatty acids (FAs), the cornerstones in lipid structures, are carboxylic acids having either a straight saturated or unsaturated aliphatic chain. FAs can be classified according to the number of double bonds in the carbon chain: saturated FAs (SFAs) with the general formula R-COOH, monounsaturated (MUFAs, with one double bond), and polyunsaturated FAs (PUFAs, with two or up to six double bonds). However, the number, position, and configuration of double bonds (cis, trans), location of branched chains, and any other structural peculiarity must be identified. Consequently, systematic terminology for FAs is troublesome for common use, and shorter options are widely used. The recommended nomenclature by the International Union of Pure and Applied Chemistry is technically clear and precise,^3^ but, for convenience, trivial or historical names are frequently used in scientific papers. O’Keefe^4^ have made an exhaustive discussion of FAs and lipids nomenclature and structure.

Lipid degradation affects a variety of products, including foods and industrial products such as lubricants. Food lipids are commonly divided into fats and oils depending on the origin of the lipid and its physical state at room temperature (RT). Fats are usually animal-based solids (lard, tallow) at RT due to their high content of SFAs. Although most vegetal lipids are found as liquids (oils) at RT, some can be found also as solids (palm, coconut) due to their high content of saturated and/or trans FAs.

In the lubricant industry, the primary role of lubrication is to reduce friction, wear, and heat between interacting surfaces in relative motion by using a lubricating substance. Traditional industrial lubricants are quite diverse. So, petroleum-based lubricants such as mineral oils, complex mixtures of C_20_–C_30_ hydrocarbons, and other compounds (naphthenic, paraffinic, and aromatic species) are used in about 90%–95% of industrial applications due to their wide range of viscosities, low cost and availability, compared to natural oils. These mineral oils may present some drawbacks such as different composition depending on the petroleum source, volatilization of low-molecular weight components, nonbiodegradability, environmental pollution (C, N, and S oxides may be emitted to the atmosphere), and hazardous waste disposal. In contrast, synthetic lubricants can be purposely developed to specific applications by well-defined chemical reactions. Due to their composition, synthetic oils are less susceptible to oxidation, to breakdown under heat, to produce unwanted byproducts, and to emulsify.^5^

In the last 25–30 years, a general wake-up to climate change started, and as result, attention is focused to the innovation and development of biobased lubricants, considered nontoxic, abundant, and easily biodegradable. There are some significant biodegradable lubricants: highly unsaturated or high oleic vegetal oils, polyalkylene glycols, low viscosity poly-α-olefins, polyol esters, and dibasic acid esters. It is true that vegetal oil-based biolubricants are more expensive than mineral lubricants, but in contrast, they exhibit unique features such as a higher viscosity index, superior anticorrosion properties, higher flash point, good lubricity, greater biodegradability and less aquatic toxicity.^6^ Thus, vegetal oil-based biolubricants appear as a promising alternative to synthetic and mineral-oil based lubricants.

Environmentally friendly lubricants, as well as the additives used in them, must fulfill the standards of biodegradation, low toxicity, health, and safety. The EU Ecolabel, established in 1992 and recognized across Europe and worldwide, is a label of environmental excellence that is awarded to products and services meeting high environmental standards throughout their life cycle from raw material extraction to production, distribution, and disposal.^7^ In this sense, at least four lubricants have already been awarded with this label: CobiolubeAgri chain (developed to respond to requests regarding the lack of ecological lubricant for the agricultural industry), CobiolubeChain (chain lubricant that facilitates a hand saw or harvester work in an exceptionally economical, convenient, and safe way), CobiolubeSawmill (developed especially for conveyors that operate outdoors) from Jarmat Oy, Finland,^8^ and NYCOLUBE 210 (designed for the lubrication of two-stroke gasoline engines running with unleaded regular fuels) from NYCO SA, Belgium.^9^

In spite of their unique functional attributes, vegetal oils possess certain limitations for their use as biolubricants such as their poor thermal and oxidative stability, that is, their low resistance to those degradation process that can change their properties and their tribological performance such as formation of undesirable species due to oxidation processes, viscosity changes with temperature, and hydrolysis due to the presence of water, as well as poor low temperature properties. This perspective describes briefly some lubrication concepts, lubrication regimes, interaction mechanisms of FAs with metal surfaces, and self-assembled monolayer formations. Special emphasis is put on the poor oxidation stability of FAs and vegetal oils and the physical and chemical approaches to improve it.

## Lubrication Concepts

### Friction, Wear, and Lubrication

Friction is the resistance force to tangential motion between two sliding or rolling surfaces in contact. The microscopic contact points between the contact surfaces are responsible for friction. In industrial applications, such as operation of mechanical systems with bearings and gears, frictional processes cause most of the mechanical energy to transform into heat with the concomitant temperature increase of the sliding bodies. These facts may have important influence on the tribological behavior of the system and failure of the sliding components. The magnitude of friction is usually expressed in terms of the coefficient of friction (COF), μ, which is the force, *F*, to slide divided by the normal load over the sliding bodies, *W*, (eq 1)
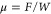
1

**Figure 1:**
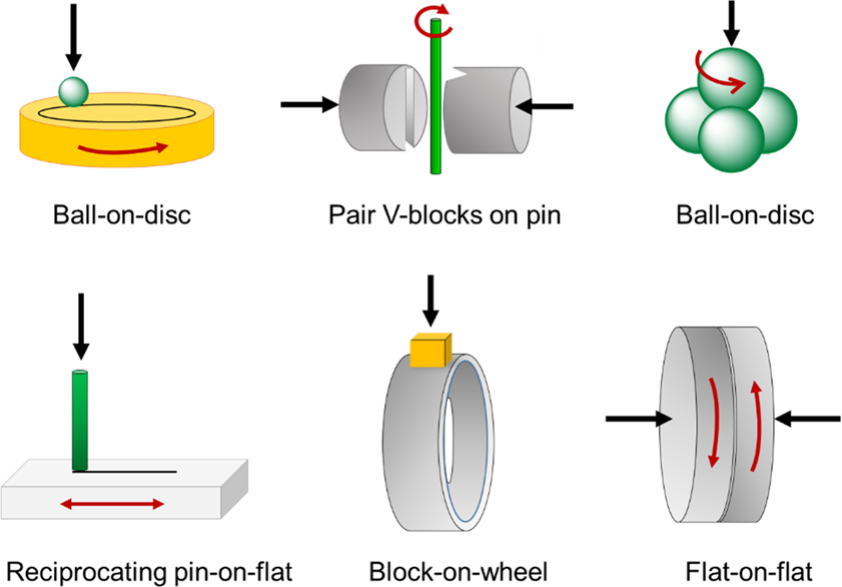
Schematic configurations of commercial tribometers. Normal load is pointed to by the black arrows.

Research in tribology makes use of commercially available tribometers, with different configurations in order to replicate real systems.^10^ Most typical configurations are depicted in Figure 1.

Excessive friction causes wear, surfaces damage, and energy losses that are not renewable. Wear is defined as the progressive material loss from the surface of one or/and other contact surface. Wear mechanisms are classified according to the type of surface damage observed on worn surfaces: abrasion, adhesion, surface fatigue, delamination, tribochemical, and so on. The wear process can be described in three steps:^11^ (a) detachment of particles from a body surface by any mechanism (adhesion, abrasion, etc.), (b) particles entrapped between the two bodies may circulate within the contact zone, creating a powder bed that keeps apart the surfaces thus reducing interactions, and (c) particles (debris) finally expelled from the contact zone while interaction between surfaces increases, wear forms, and the cycle begins again.

### Lubrication Regimes

**Figure 2:**
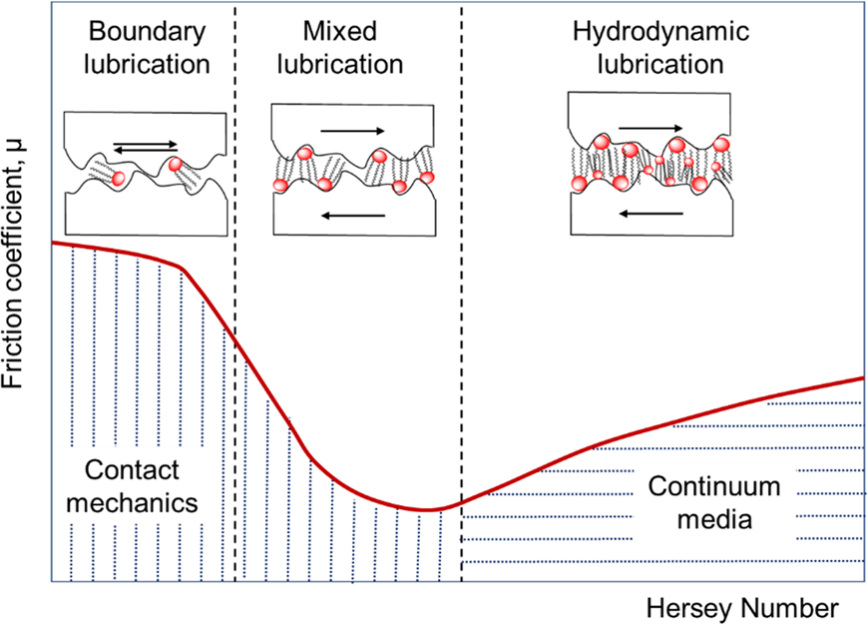
Stribeck curve illustrating different lubrication regimes and relations to coefficient of friction, speed, and lubricant viscosity.

Oil-based lubricants may be used to provide a protective layer that reduces/minimizes the frictional force and wear between contacting surfaces in motion. The magnitude of the normal load between the surfaces in contact is responsible for different lubrication conditions or regimes: boundary, mixed, hydrodynamic, and elastohydrodynamic (EHL). The Stribeck diagram (Figure 2), a plot of a lubricated contact’s friction coefficient vs the Hersey number, explains the different lubrication regimes. The Hersey number is a dimensionless function of all three tribological test conditions: sliding velocity and pressure, as well as temperature by means of viscosity η (*T*).

For high values of the Hersey number, the COF increases linearly due to viscous dragging forces in the oil film. However, when the load increases or sliding velocity and/or viscosity decreases, the Hersey number falls. In these conditions, the wedge or layer of lubricant oil is sufficiently thick (normally >1 μm)^12^ to avoid the opposite surfaces coming into contact. This situation, the hydrodynamic lubrication (HDL) regime, provides very low friction and a high resistance to wear. The elastohydrodynamic lubrication (EHL) is a particular form of HDL in which elastic deformation of surfaces and piezoviscous effects become significant. For even smaller values of the Hersey number, the lubricant film is further reduced. If the film thickness and the surface asperities are of similar dimensions, contact between the surfaces starts, and the COF increases as the Hersey number decreases. In these conditions of lower speed, higher load, or higher temperature, the lubricant viscosity decreases, and some asperities occasionally come into contact. The lubrication regime is termed mixed lubrication regime. Further reduction of the Hersey number results in a boundary lubrication regime in which most asperity contacts between the surfaces are due to a thinner lubricant film. Friction and wear under boundary conditions are the most severe as most of the load rests on asperities in physical contact.

Vegetal-based oils, as functional fluids (ester), are liquids at RT and can be used in all the regimes. Particularly, vegetal oils are effective as boundary lubricants due to the FA composition that allows strong interactions with the lubricated surfaces forming self-assembled tightly packed monolayers which provide ultralow friction.^13,14^ The formation of self-assembled monolayers of FAs on metal surfaces is a complex process in which physical adsorption, chemisorption, and tribochemical reactions on the surfaces may take place. Physical adsorption involves intermolecular forces (hydrogen bonds, van der Waals), and as no particular chemical functional groups are needed, all FAs may potentially form such layers. On the other hand, chemisorption involves sharing of valence electrons between the oil and the surface and can be an irreversible or partially irreversible mode of adsorption. Tribochemical reactions deal with the ability of vegetal-based oils to undergo chemical reactions by themselves or with other materials (water, oxygen, metal) in the friction zone. The adsorption mechanisms of FAs on materials of industrial interest have been addressed from different points of view, due to their efficacy to protect surfaces in the boundary lubrication regime (high load, low sliding velocity) and also as friction modifiers of base lubricants. In practice, under boundary lubrication, different factors may influence the film structure and its protective tribological properties.

### Nature of the Substrate Material

Different sliding surfaces have been used to study the boundary lubrication mechanisms of FAs, as the base components of vegetal-based oils. It seems that FAs attach to metal oxide surfaces through the carboxylate headgroup, while the long alkyl chains may be involved in intermolecular van der Waals interactions, which provide not only the molecular organization of the layer but also induce a high packing density (SAM, self-assembled monolayers). As most common metals are reactive, chemisorption is the form in which FAs interact with them. The strength of the chemical bonding between FAs and the metal surface depends on the reactivity of the metal.^15^ According to Bowden et al.,^15^ on Zn, Cd, and Cu surfaces, the percentage of a retained monolayer of lauric acid after washing was notably high in comparison with the poor retention of metals such as Mg or Cr. This study suggests that a reaction of lauric acid with the substrate has occurred for the former group of metals.

Tao^16^ showed that the metal substrate nature dominates the binding geometry of the headgroup and most likely the packing density. So, chemisorption of *n*-alkanoic acids, CH_3_(CH_2_)_*n*_COOH, *n* = 2–18, on AgO surfaces involved the carboxylate group binding in a nearly symmetrically mode, while on the surface of Al_2_O_3_ and CuO the binding was asymmetric with tilt angles estimated between 15° and 25° from the surface normal. Also, infrared studies suggested that monolayers of *n*-alkanoic acids on AgO were more ordered than their counterparts on Al_2_O_3_. However, Raman studies^17^ suggested just the contrary; a monolayer of stearic acid adsorbed to a smooth AgO surface is less ordered that the stearic acid layer on Al_2_O_3_.

Despite the considerable research, with the chemisorption of FA on metal surfaces under boundary lubrication, the monolayer morphology is unclear due to the complexity of the interfacial processes involved. Ratoi et al.^18^ using ultrathin film interferometry to monitor the lubricant film thickness during rolling contact of bearing steel on glass observed that carboxylates of metals below iron in the electrochemical series (e.g., Cu, Pb) reacted to form thick iron carboxylate boundary films, while carboxylates of metals above iron (e.g., Zn, Al, Ca, Mg) did not form boundary films at all. Lim et al.^19^ have also explored the mechanism of stearic acid bonding on amorphous aluminum oxide (alumina) and on single-crystal C-plane aluminum oxide (sapphire) surfaces. Using X-ray photoelectron spectroscopy (XPS) confirmed the presence of aliphatic and carboxylic groups, while infrared spectroscopy provided key information about the different binding mode of the carboxylic acid head and the aluminum oxide surfaces; stearic acid binds to sapphire surfaces via a bidentate interaction of carboxylate through two oxygen atoms, while both bidentate and monodentate interactions may take place with alumina surfaces. Recent investigations by Simič and Kalin^13^ using atomic force microscopy (AFM) explained the monodentate form of FAs on a steel surface as due to the dissociation of the carboxylic acid into a carboxylate anion and a proton, possible due to a chemical reaction between the carboxylate anion and the surface metal atoms (typically positively charged). In the bidentate configuration the carboxylate group is bound to the surface by both oxygen atoms.^20^

**Figure 3:**
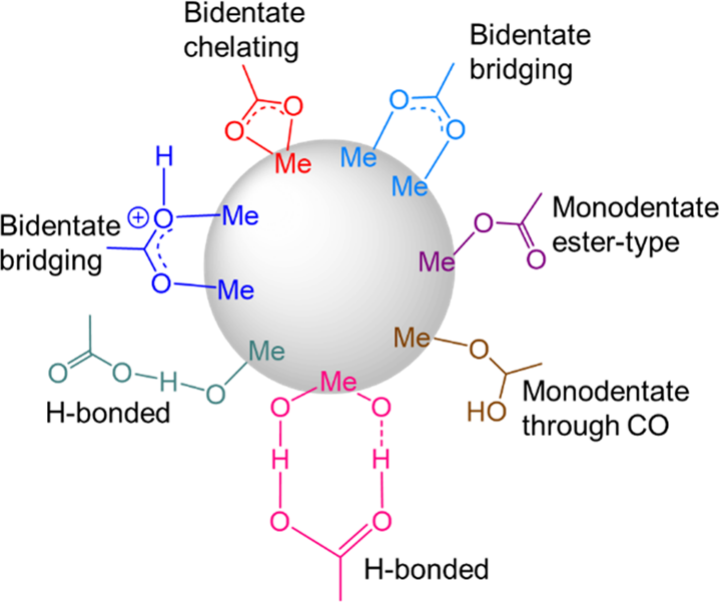
Interaction modes of the carboxylic group with the surface of a metal oxide/hydroxide nanoparticle.

Similar models also have been applied to the boundary lubrication at the nanoscale, a field of interest not only from a basic point of view but also due to the increasing demand in understanding the lubrication behavior of ultrathin lubricant films on smooth solid surfaces, particularly in high-density magnetic recording technology and micro/nanoelectromechanical systems (MEMS/NEMS).^21^ Over the past two decades, nanomaterials have been investigated and employed in tribological applications, in particular, in the development of efficient “nanolubricants” in which nanoparticles of metal oxides, metal sulfides, metals powders, and carbon-based nanomaterials have been of special interest.^22,23^ In fact, inorganic and carbon-based nanoparticles have been demonstrated to be promising additives due to their noteworthy capacity to reduce friction and wear and to improve oxidative stability. The main drawback of inorganic nanoparticles as lubricant additives is their poor ability to form a stable suspension in oil. Consequently, to tackle this obstacle, adequate surface modification of the nanoparticles has to be done. In this sense, vegetal oils have the capacity to provide the nanoparticle surface with a hydrophobic layer, compatible with the base lubricant thanks to their FA content. FAs bind strongly to surface metal oxide nanoparticles, and they are then an excellent choice as modifying agents. Among them, oleic acid, having a C18 tail with a cis double bond, is one of the most used, as it can form the kinks necessary for effective stabilization. Oleic acid provides the surface of a dense protective monolayer that renders nanoparticles in a highly uniform size range. In Figure 3, carboxylate binding modes proposed by Galoppini^24^ have been recreated on the surface of a metal nanoparticle.

### Fatty Acid Unsaturation and cis/trans Conformation

The FAs unsaturation degree has only a marginal effect on the chemisorption of FAs to the substrate as this process depends mostly on the interaction of the carboxylic headgroup and the substrate nature. However, the physically adsorbed amount and friction have been found to increase with the unsaturation degree of FAs. Campen et al.^25^ have demonstrated that in the boundary lubrication regime the presence of stearic acid adsorbed on steel surfaces resulted in a COF that increased with log(speed), characteristic of close-packed vertically oriented monolayers with linear configuration. Elaidic acid, the trans isomer of oleic acid, gave the same trend as stearic acid. However, oleic acid gave a COF constant over the speed range assayed. Oleic acid, due to its cis arrangement, could not adopt a linear configuration and thus could not form close-packed monolayers. Consequently, the structure of the adsorbed film is dictated by the molecular structure of the FAs, which, in turn, may have a quantitative effect on the COF. On the other hand, the degree of molecular interaction plays an important role in boundary lubrication. The molecules of saturated FAs, like stearic acid, may align themselves in straight chains and be closely packed on the surface providing a strong protective layer. The presence of double bonds in FAs hinders rotation and pushes the chains to bend, resulting in a loosely packed monolayer with poor protective action.^26^ Bowden and Tabor^27^ took an important step forward in the understanding of boundary lubrication studying the effect of the number of stearic acid layers on stainless steel surfaces by means of the Langmuir–Blodgett (LB) technique. It could be observed that the lubrication effectiveness increased with increasing the number of films. The study also demonstrated that while a single monolayer of a stearic acid could reduce friction, the layers were not robust and required continuous replenishment.

### Fatty acid chain length

The effectiveness of a boundary FAs lubricant depends on its chain length. Results from Castle and Bovington,^28^ using a series of long chain carboxylic acids, have shown that measured boundary COFs decreased with increasing chain length (C12 to C24) and unsaturation level (0 to 3 inclusive). Also, it was shown that the durability of boundary FAs films increases with molecular weight in the range of 18–26 carbon atoms due to a stronger cohesion among the chains.^29^

### Tribochemical reactions

**Figure 4:**
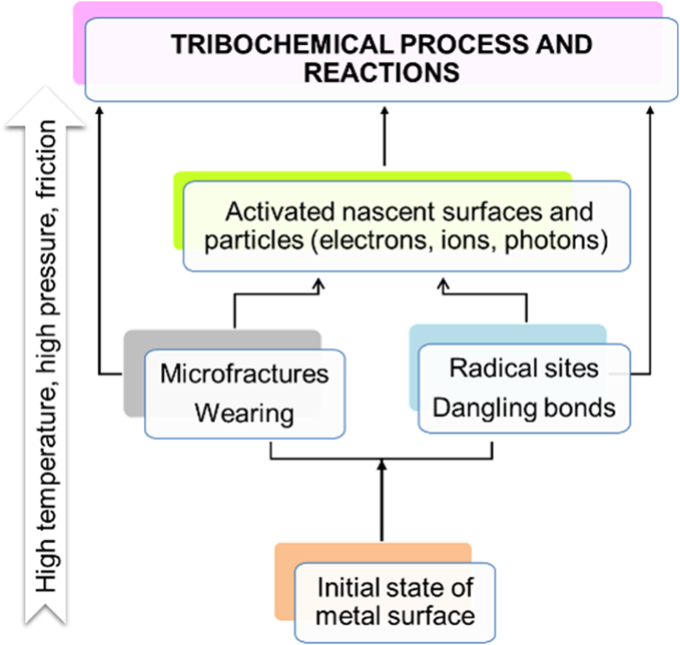
Processes associated with surface physical and chemical changes during friction.

Under boundary lubrication regime, interaction between asperities results in high friction and severe wear, which encompass high temperature, triboemission and tribochemical reactions.^30^ Triboemission is defined as an emission of electrons, charged particles, lattice components, photons, etc., under conditions of boundary friction conditions and/or surface damage caused by fracture processes. Figure 4 illustrates the triboemission process associated with the surface changes during friction.

Under a boundary lubrication regime, a high mechanical energy is developed, and it can be easily dissipated as thermal, chemical, optical, and electrical energies.^31^ A tribochemical reaction is a chemical reaction initiated by the absorption of mechanical energy which acts on the solid surfaces forming active sites. Two types of activated sites can be generated upon friction: thermally activated sites and low-energy electron-activated sites. The low-energy electrons (exoelectrons) are spontaneously generated electrons (0–4 eV) from raw surfaces and/or emitted from activated surfaces. According to the model by Smith and McGill,^32^ with friction, metal–metal and metal–oxygen bonds are broken. The cleavage of the metal–oxygen bond produces electrons and leaves positively charged sites and an oxygen dangling bond on the surface. The electrons then interact with a water molecule releasing a hydrogen radical (H^•^) and a hydroxi anion (OH^–^). Subsequently, the hydroxide groups neutralize the FAs, thus resulting in the formation of water and the corresponding carboxyl anions. Simultaneously, the hydrogen radicals combine themselves to form hydrogen molecules. Finally, the fatty carboxyl anions react with the surface positively charged metal cations to form soap. Upon friction, the monolayer structural integrity may stay constantly damaged by either chemical or tribochemical reactions within the contacting surface, all of which leads to the developing of thin coatings (tribofilms) that affect friction and wear behavior. The properties and thickness of the tribofilms are quite different from the starting monolayer and once formed and adhered to the surfaces may govern the tribological performance of the sliding contacts.

### Achilles Heel of Vegetal Oils as Lubricants: Auto-Oxidation

Vegetal oils offer a great potential for developing innovative environmentally friendly lubricating products. Most of vegetal oils consist primarily of triacylglycerols (TAGs) (∼98%),^33^ the composition of which is specific to the origin of each oil. Most of the FAs in TAGs are straight chains of an even number of carbon atoms, in the range of C_4_–C_22_. Natural FAs up to C_18_ are typically fully SFAs, with unsaturation or polyunsaturation being more common for higher carbon numbers. Some other minor components in vegetal oils include mono- and diacylglycerols (MAGs and DAGs), free fatty acids (FFAs), phosphatides, sterols, tocopherols and tocotrienols, pigments, and fatty alcohols.^34^

**Figure 5:**
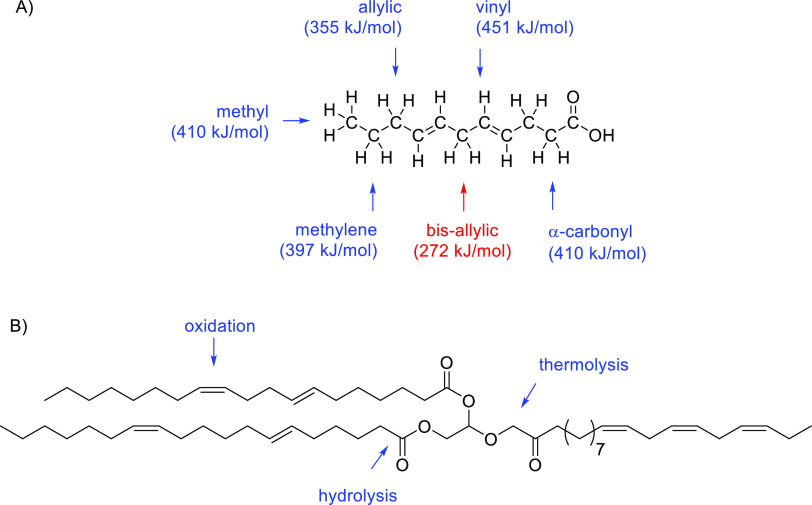
(A) Structure showing the dissociation energies (kJ/mol) of C–H bonds in fatty acids. (B) Physically and chemically attackable domains in TAGs.

Composition of biodegradable lubricants is particularly important in relation to their physical and/or chemical degradation. So, FFAs and FAs in triglycerides have the ability for hydrogen abstraction as result of the presence of methylene and/or bis-allylic hydrogens with low bond dissociation energies (Figure 5A). On the other hand, in vegetal oils, fatty acids have mostly cis conformations with the content of trans fatty acids lesser than 4%.^35^ The trans-configuration is thermodynamically more stable than the cis. For example, for linoleic acid (C18:2), the C–H bond dissociation energy in the configuration C18:2-9c-12c is 328.0 kJ mol^–1^, while it is 334.8 kJ mol^–1^ is for the C18:2-9t-12t configuration, a difference of 6.8 kJ mol^–1^ which indicates that oxidation takes place preferentially at cis double bonds.^36^

**Figure 6:**
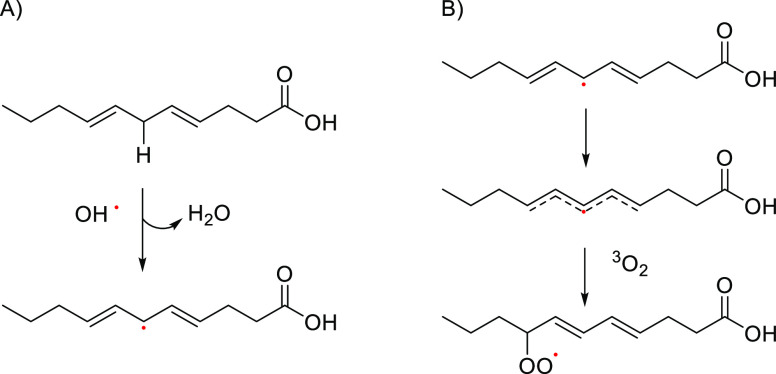
(A) Initiation process of lipid oxidation with the reactive oxygen hydroxyl radical. (B) Resonance system with formation of conjugated dienes and addition of oxygen to yield hydroperoxide radicals.

Also, different polar bonds in TAGs are active sites making them easily attackable by humidity, oxygen, and other chemical and physical agents (Figure 5B). In particular, one of the main weakness is the degrees of unsaturated double bonds that function as active points for many reactions, with autoxidation common to TAGs and FFAs. The mechanism of autoxidation of vegetal oils, a free radical chain reaction, is well known and includes three steps: initiation, propagation, and termination. The process is initiated by formation of TAGs or FA radicals and the most commonly form of atmospheric triplet oxygen, ^3^O_2_, a radical with two unpaired orbitals in the molecule. However, this interaction is thermodynamically unfavorable due to different spin states unless oxygen is activated and converted to singlet oxygen (^1^O_2_) or to a reactive oxygen species such as hydrogen peroxide (H_2_O_2_), hydroxyl radical (OH^•^), or superoxide anion radical (O_2_^•–^). An input of energy (heat, light, radioactive radiation) or the presence of natural sensitizers present in the oil may mediate the activation of oxygen. The initiation auto-oxidation step occurs with the abstraction of hydrogen atoms from TAGs and FAs, in the presence of reactive oxygen species (Figure 6A).

The hydrogen abstraction occurs preferentially at allylic hydrogens with the weakest C–H dissociation energies. As seen in Figure 6A, the abstraction of the hydrogen atom in the methylene group between two double bonds in FA is by far the most readily removed as only 272 kJ mol^–1^ are necessary. The reactivity order is then as follows: doubly allylic H ≫ singly allylic H > H α-carbonyl > H methylene farther down the acyl chain. The hydrogen abstraction leaves a carbon-centered lipid radical (R^•^) which is stabilized through a resonance molecular rearrangement (Figure 6B). The (R^•^) so formed reacts then with ^3^O_2_ to form new radicals, the reactive alkyl peroxy radicals (R–O–O^•^) that can then remove hydrogen from another lipid molecule and react with hydrogen to form hydroperoxides (R–O–OH) and new free radicals (R^•^). As the reaction of peroxyl radicals (R–O–O^•^) with the chain FA (RH) is slower than that of RH with ^3^O_2_, that reaction determines the autoxidation reaction rate. Hydroperoxides may also break down to produce more free radicals, propagating the oxidation process. This second stage of the autoxidation process is the propagation step, and the succession of reactions can be repeated several times due to the proliferation of radicals. The termination of the free radical chain reactions takes place when the FA substrates diminish and the reaction among alkyl and peroxyl radicals combine to give rise to many nonradical volatile and nonvolatile products.

Little work has been published about the impact of oxidation on the lubrication performance of vegetal oils. For the primary oxidation compounds of TAGs-based vegetal oils, Fox et al.^37^ have shown that an increase in the hydroperoxides content during sunflower oil oxidation resulted in wear and friction increase, although it was uncertain which, the degradation of TAGs or the hydroperoxides themselves, had the higher impact. Also, during the lubrication process, hydroperoxides may be further degraded liberating not only small molecules but also condensation and/or corrosive products that subsequently increase the oil viscosity and/or may form sediments by polycondensation, all of which impair the lubricant properties of the oils. So, the higher the lubricant viscosity is, the greater the force against its resistance to flow between devices is. The small molecules liberated during decomposition of hydroperoxides (secondary stage of vegetal oil oxidation) may be volatile (e.g., aldehydes) and nonvolatile (e.g., short-chain hydrocarbons, alcohols, epoxides) compounds. Due to the low polarity and short chain length of volatile compounds, their impact on lubrication must be marginal. However, the polymerization of the high molecular weight compounds formed during the final stages of the oxidation process may lead to an increased viscosity and the formation of lacquer deposits, the two factors associated with engine damages.

### Photo-Oxidative Degradation of Vegetal Oils

In the presence of UV light, another important oxidation pathway may accompany the auto-oxidation reaction, chlorophylls act as photosensibilizers while in the dark they act as antioxidant compounds. Upon absorption of light energy, chlorophylls are excited to a singlet excited state from which the triplet state can be populated through a nonradiative intersystem crossing process. Triplet state chlorophylls react then with triplet oxygen producing singlet oxygen by energy transfer, thus chlorophylls returning to their ground singlet state.^38^ Singlet oxygen can react directly with electron-rich double bonds giving rise to conjugated and nonconjugated hydroperoxides (“ene” reaction), and no alkyl-radicals are formed. It is worth stressing the different roles of oxygen in auto-oxidation (triplet oxygen) and photo-oxidation (singlet oxygen):^39^ (a) Triplet oxygen does not directly react with double bonds, but singlet oxygen does. (b) Singlet oxygen oxidation proceeds through an Alder-ene reaction instead of a radical chain one. (c) The singlet oxygen reaction is quicker, and its rate is related to the number of double bonds instead of types of double bonds (conjugated or nonconjugated). (d) The reaction does not depend on the number of doubly activated allylic groups. Hydroperoxides formed by ^1^O_2_ oxidation are decomposed by the same mechanisms that the hydroperoxides are formed by ^3^O_2_ in auto-oxidation, and the drawbacks for lubrication are also the same as already mentioned.

### Thermo-Oxidative Degradation of Vegetal Oils

Thermal stability is a critically important lubricant characteristic. During the lubrication process, mechanical energy is transformed into heat, which results in the increase of the temperature of the sliding bodies, particularly within the contact region where the temperature is highest.^40^ So, most neat vegetal-based lubricants can only be used in low-performance applications with low thermal stress as the maximum operating temperature is restricted to 70 °C.^41^ On the other hand, at low temperatures, depending on both the plant species and the strain from which the oil was obtained, vegetal oils may exhibit poor cold flow behavior.

Although the temperature effect on the oxidative stability of vegetal oils has been studied for many years, the true reaction mechanisms of the thermal decomposition of TAGs is not clear due to its complexity. In studies performed by Nawar’s group,^42−44^ FFAs are thermally formed both in the presence and absence of moisture, with a release of shorter-chain and unsaturated FAs. This means that the oil becomes more acidic during degradation.

Bond cleavages may occur in any point of the TAG structure, with each scission producing a large variety of products (lactones, methyl ketones, hydrocarbons, and many other compounds) and free radicals that may initiate lipid oxidation chain reactions. At high temperatures, as a result of the degradation, the oil loses its lubricating properties (increase in lubricant viscosity), while the final products of thermal degradation eventually combine with each other to form oil–insoluble polymeric materials as sludge (insoluble in the bulk oil) or as varnish or lacquer (on metal surfaces).^45,46^ The poor thermo-oxidative properties of vegetal oil lubricants are mainly due to the following: (a) Hydrolytic instability of the β-CH group on the glycerol group (Figure 5) that results in the ester linkage decomposition into FFAs and alcohols. The hydrolytic reaction rate increases at elevated temperature. (b) The temperature properties of vegetal oil-based lubricants are mainly dependent on the amount of PUFAs. Polyunsaturation leads to oxidation and polymerization reactions through a complex process that involves several short-lived species and several intermediate steps. (c) The presence of metals (e.g., iron, copper) catalyze the initiation of the oil degradation at much lower temperatures.

### Metal Ion-Mediated Degradation of Vegetal-Based Lubricants

Crude vegetal oils may contain transition metals such as copper or iron, particularly in chelated form.^47^ Without refining, vegetal oils contain relatively high amounts of copper and iron.^48−50^ These metals reduce the activation energy of the auto-oxidation process and may both initiate and catalyze the first degradation step producing reactive ^1^O_2_ species from ^3^O_2_ and hydroxyl radicals from hydrogen peroxide (Haber–Weiss reaction), while H_2_O_2_ can also form hydroxyl radicals by the Fenton reaction in the presence of Fe^2+^.^48,51^ Also, metal ions (Cu^2+^ or Fe^3+^) can catalyze the decomposition reaction of hydroperoxides to alkoxy-radicals, which may amplify and/or initiate *de novo* the lipid peroxidation. Fe^2+^ is more active than Fe^3+^ in decomposing hydroperoxides, with a reaction rate constant of 1.5 × 10^3^ M^–1^ s^–1^.^52^ According to Choe and Min,^48^ copper accelerates hydrogen peroxide decomposition 50 times faster than ferrous ion (Fe^2+^), and ferrous ion acts 100 times faster than ferric ion (Fe^3+^).

### Moisture Contamination

Unlike mineral oils, vegetal oils exhibit much higher water solubility. In order to set limits on moisture levels, the concept of relative saturation is useful. So, if 10 ppm moisture is considered adequate for a dry mineral oil (moisture saturation at 60 ppm at room temperature), then for a vegetal oil (1200 ppm water saturation at room temperature) the equivalent water content would be around 200 ppm. The concentration of water may vary from 300 to 400 ppm for mineral oils, while for synthetic biodegradable oils it ranges from 800 to 1000 ppm.^53^

When dispersed in a lubricant, moisture is a contaminant that reduces the lube chemical stability and may damage the bearing surfaces. Water may enter the lubricant in different ways, such as absorption, condensation, combustion/oxidation/neutralization, and free water entry, and it can be present as dissolved, emulsified, and free water. The moisture from room air humidity in constant contact with the lubricant represents the lowest level of moisture contamination, the dissolved moisture. Up to a relative humidity of air of 100%, water molecules are dispersed, do not condense, and are not visible to the naked eye. If temperature decreases to a point that water molecules condense, then the lubricant becomes milky like (emulsified water). Free water is water that remains as a separated aqueous liquid phase in the lubricating oil. For a tribo-system, dissolved water is less damaging than emulsified water, while free water is the most damaging.^54,55^ Direct effects of water contamination in lubricants causes many problems to machinery life such as corrosion (chemical or electrochemical reaction between the metal surface and water),^56^ water etching (due to byproducts from lubricant degradation), vaporous cavitation (due to instantaneous vaporization and condensing implosion of water),^57^ and hydrogen embrittlement (under extreme conditions water dissociates to release hydrogen atoms which then permeate the metal causing pinning and embrittlement of the metal),^58^ among other effects. Water contamination also has undesirable effects on the physical and chemical properties of the vegetal oil itself. As described above, the oxidation process in oils begins with an initiation step that requires a catalyst (metal ions, light, temperature, oxygen) and the accumulation of ROOHs responsible for propagation of the oxidation. The last 10 years have seen the emergence of new hypotheses to explain the water role in the oxidation process in oil related with the formation of reverse micelles: lipid hydroperoxides, due to their amphiphilic nature, self-assemble into roughly spherical arrangements (reverse micelles) around trace amounts of water, placing the hydrophilic head (e.g., carboxyl, phosphate, polar groups) toward the water domain (aqueous core) and their hydrophobic tails (hydrocarbon chains) toward the bulk oil.^59^

To date, there are convincing observations and excellent reviews that support the hypothesis that reverse micelles and/or lamellar structures are the natural nanoreactors where lipid oxidation takes place.^60−63^ In the presence of water contamination (along with the activity of lipase enzymes), hydrolysis of TAGs takes place, producing MAGs, DAGs, and FFAs, compounds that have been reported also as pro-oxidants in several works, in particular FFAs.^64,65^ A proposed mechanism for the pro-oxidant activity of minor components in oils (FFAs, MAGs, phospholipids) is based also on their amphiphilic nature. Reverse micelles are formed by amphiphilic molecules with a low hydrophilic–lipophilic balance (HLB), as is the case for minor components of oils: FFAs (HLB ≈ 1.0), DAGs (HLB ≈ 1.8), and MAGs (HLB ≈ 3.4–3.8); however, phospholipids have intermediate HLB values (HLB ≈ 8.0), the reason that they form not only reverse micelles but also lamellar structures.^66^ Subramanian et al.^67^ reported the presence of reverse micelle structures in crude soybean oil and high-oleic sunflower oil containing 245 and 400 ppm water, respectively. The presence of reverse micelles in bulk oils creates oil–water interfaces where hydrophilic (e.g., metal ions) and amphiphilic (e.g., lipid hydroperoxides) pro-oxidants are driven into close contact with each other, resulting in increased lipid oxidation rates.^68,69^ What has not been supported by experimental data is the fact that if hydroperoxides have an active role in the formation of reverse micelles, then these compounds should exhibit a critical reverse micellar concentration. According to Brimberg and Kamal-Eldin,^70^ lipid oxidation starts by pseudo-first-order slow buildup of hydroperoxides until reaching a critical concentration that triggers the oxidation of the oil passing from the induction to the propagation phase and the reaction rate change to a second-order reaction. The increase of reverse micelles containing hydroperoxides, water, and other amphiphilic molecules provides an interfacial nanoenvironment within the oil, acting as active mediators for oxidation reactions. As the concentration of hydroperoxides and water increases (as result of oxidation reactions), micelles become larger until a critical point upon which they disrupt and smaller micelles can be formed. Although there is much information about the deleterious tribological effects of water contamination in conventional and synthetic (bio)lubricants, there is not similar information published for vegetal oil-based lubricants yet.

### Effect of Lubricant Oxidative and Thermal Degradation upon Viscosity

Viscosity is one of the most important physical properties of a lubricating oil as it plays a decisive role in the formation of an oil film at the interface between rubbing surfaces. As a result of oxidative and thermal degradation, a decrease in the oil viscosity should be expected due to the formation of different low molecular weight compounds and FFAs. However, most analytical tests reveal an increase in viscosity during the auto-oxidation and thermo-oxidative processes. This fact has been explained considering that when oil starts to oxidize, the amount of carboxylic acids increases. These acids are effective catalysts for aldol condensation reactions of aldehydes and ketones, thus converting the low-molecular weight carbonyl compounds into higher molecular weight oligomers and low molecular weight polymers, which are responsible for the increased viscosity.^71^ As the reaction proceeds, insoluble oligomers, sludge, and varnish deposits are formed, ruining the lubricant oil performance.

## Stabilization of Vegetal Oils

As seen above, vegetal oils are easily degradable, with this property both an advantage (e.g., environmentally friendly lubricants) and a drawback (e.g., limited direct lubrication applications due to poor oxidative/thermal stability). To overcome/minimize such weak points several chemical modifications can be performed.

### Inhibitors of Oxidation

Some compounds, sulfur-based and aromatics, can retard/inhibit the oxidation of vegetal oils by radicals and peroxyl radicals, extending their useful lifetime. As the oxidation inhibitors are consumed during lubrication, they must be used in most lubricant applications. There are different types of compounds to interrupt the oxidative degradation of oil lubricants:

(a) *UV absorbers*. This type of compound protects oils against the effects of light, which is photo-oxidation (singlet oxygen quenchers). As this process does not involve radicals, no other antioxidants can perform the task. UV absorbers inhibit the photo-oxidation initial step due to their absorptivity, much higher than that of the vegetal oil chromophors. The UV energy absorbed is dissipated as heat. A problem with some absorbers is related with its secondary function as free-radical terminators, a process during which the absorbers are consumed. So, a compound purposely designed for free-radical scavenging should be added to conserve the absorber for its true function. Typical examples of vegetal UV absorbers are carotenoids, such lycopene, lutein, and zeaxanthin.
(b) *Peroxide decomposers*. Also known as high-temperature antioxidants, this class of antioxidants decompose, via a nonradical path, hydroperoxides and peroxides as soon as they are formed during the propagation stage of the chain reaction. The products of decomposition are molecular compounds. The propagation is thus interrupted and ends in stable products. Common peroxide decomposers include trialkylphosphites and simple aromatic sulfur compounds. Most of these compounds also exhibit both antiwear and anticorrosion properties.
(c) *Chain-breaking antioxidants*. This is the most important and effective group of antioxidants that inhibits/retards the radical propagation chain. Thes types of antioxidants scavenge the initial free radicals by two mechanisms: as electron acceptors (oxidizing agents) and electron donors (reducing agents). The final stable compounds are phenoxyl radicals that are unable to propagate the chain reaction. Examples of these antioxidants are α-tocopherol (vitamin E) and flavonoids. An important characteristic of chain-breaking antioxidants is their synergistic behavior when used in combination with other coantioxidants. For example, flavonoids may act in synergy with α-tocopherol.^72^
(d) *Metal deactivators*. In this class of oxidation quenchers, nitrogen compounds convert metal ions into catalytically inactive chelates. Typical examples include Schiff’s bases thiadiazoles, oxamides, curcumin, phytic acid, and quercetin.

### Water–Oil Interfaces and Antioxidants Effectivity

The effectiveness of antioxidants is a topic that has attracted much attention, and often data have been explained taking into account (a) the hydrophobic–hydrophilic nature of the antioxidant and (b) extrapolating the data obtained in bulk oils to lipid dispersions. In the milestone work by Porter et al.,^73^ it was observed that hydrophilic antioxidants were more effective than lipophilic antioxidants in bulk oils, a fact that resulted paradoxically in the reason for which they named such behavior as the “polar paradox”, while no mechanisms or explanations for it were postulated. Later, in other breakthrough publications by Frankel et al.,^74^ the polar paradox was explained as due to an interfacial phenomenon and partition in the media. So, in bulk oils, hydrophilic (polar or partially fat-soluble) antioxidants have the ability to orientate themselves at the air–oil interface where surface oxidation occurs, thus protecting the system from oxidative changes, whereas hydrophobic antioxidants are more effective in relatively more polar media such as oil-in-water emulsions. At the same time, Koga and Terao^75^ refused the hypothesis that the air–oil interface was the lipid oxidation site in bulk oils arguing that as air (dielectric constant = 1) is less polar than edible oils (dielectric constant = 3), any driving force would impel polar antioxidants to move toward the air–oil interface. In their study, they reported that α-tocopherol was an efficient free-radical scavenger in bulk oils containing a small amount of water (1%) due to its increased partitioning within the oil–water interface. More refined theories about the interfacial phenomenon followed including association structures such as reverse micelles and lamellar structures.^60^ Many experimental works followed contradicting the polar paradox, as revisited in excellent reviews.^76−78^

Experimental studies in oil–water dispersions demonstrated that the antioxidant properties were governed by their HLB, which determine not only their partition in association structures such as reverse micelles and lamellar structures but also their ability to self-aggregation and their interaction with oil minor components. In fact, oil nature also affects emulsion properties (e.g., size, viscosity, and stability) and the accessibility of the antioxidants and other bioactives incorporated.^79^ Studies carried out by varying the hydrophobicity of antioxidants through esterification with alkyl chains of different lengths showed that esterified phenolic antioxidants obeyed the polar paradox hypothesis up to a critical point, C_8_–C_12_ chain length, beyond which a sudden decrease of antioxidant activity was observed. This fact has been named as the “parabolic effect” and more recently as the “cutoff effect”. The first mention of this effect was made by Laguerre,^80^ and it is related to the molecular size of antioxidants showing low mobility due to steric hindrance which decreases their diffusibility toward the interface. Too-short or too-long hydrophobic chains in a homologous series of antioxidants do not guarantee an optimal antioxidant activity; below a given hydrophobicity threshold, antioxidants are located in the aqueous phase, not close enough to the lipid–water interface where oxidation takes place. However, when the hydrophobicity threshold has (critical medium-sized chains) antioxidants concentrated at the lipid–water interface, oxidation is efficiently hampered. Recently, Mitrus et al.^81^ investigating the effects of gallic acid and some of its alkyl derivatives on the oxidative stability of soybean O/W emulsions demonstrated that antioxidants accumulate in the interfacial region, where the effective concentration is 20–180 times higher than the stoichiometric concentrations. Beyond the cutoff point, the antioxidants are far from the interface and located within the bulk oil.^82^ In summary, there is an optimum point (cutoff effect) for each antioxidant that depends *inter alias* on its HLB and its concentration. Although much of the work devoted to the role of antioxidants within the interfaces in W/O and/or O/W emulsions in the field of foods, to our knowledge, such information is scarce if not missing for vegetal-based lubricants, as well as the relationship between the presence of reverse micelles in the W/O emulsions and the cutoff effect.

### Natural Antioxidants

Due to increasing concern over environmental issues, the conventional antioxidants used in mineral oil-based lubricants should be replaced by others that are environmentally acceptable. Stabilizing vegetal oil-based lubricants under boundary lubrication conditions can be challenging due to the extreme conditions of temperature, friction, water, and oxygen, not only because the rate of the oil oxidation is quite high but also because the thermal stability of the antioxidant may be compromised. Notwithstanding, there is increasing interest in vegetal-based antioxidants to replace the applications of synthetic ones. There are many types of compounds with antioxidant properties that can be found in natural sources such as seeds of many fruits, vegetables, cereals, aromatic plants, and olive oil, among others. Natural antioxidants can function as singlet and triplet oxygen quenchers to inhibit photo-oxidation and auto-oxidation, respectively, as well as free radical scavengers and peroxide decomposers. Most of these natural antioxidants are phenolic compounds, which can be broadly classified into two classes: flavonoid and nonflavonoid polyphenols. At the same time, flavonoid polyphenols can be divided into different subclasses as a function of the degree of unsaturation and oxidation of the heterocyclic ring: anthocyanins, flavonols, flavanones, flavanols, flavones, and isoflavones.^83^ Flavonoids and stilbenes are the largest group of polyphenols and may act as chain-breaking peroxyl radical scavengers. Nonflavonoid antioxidants include ascorbic acid, plant pigments, carotenoids, and tocopherols. Carotenoids are the largest group of terpenes and function as singlet oxygen quenchers. The food industry has investigated the oxidative stability of vegetal oils for years, and despite that much of the research cannot be directly applicable to the tribology field, some of the fundamental principles are the same. So, much work devoted to the use of plant extracts containing phenolic compounds has been carried out due to their functional and nutritional effects, including antioxidant activity.

## Modifications of Vegetal Oils to Improve Oxidative Stability

Selection of the most effective natural antioxidants for vegetal oil lubricants is not enough to rival the oxidative stability provided by mineral oil-based lubricants. Also, raw vegetal oils have chemical properties not suitable for specific lubrication applications due to their high saturated or PUFAs content, their low volatility, and high viscosity. Some of these properties can be modified by several approaches such as the following:

- *Blending*. Blending has been tested to improve the oxidative stability of vegetal oils. Li et al.^84^ studied the blending of SBO with sea buckthorn, camellia, rice bran, sesame, and peanut oils (20% v/v), observing that the oxidative stability of oil blends was higher than that of the raw soybean oil. It was ascribed to the change in the FAs and tocopherols profiles and the minor bioactive lipids present in the selected oils. Recently, Ali et al.^85^ also observed that blends of SBO with rice bran oil (60% v/v) improved the oxidative stability of SBO under extreme thermal conditions (170 °C, 12 h heating) thanks to the significant amounts of tocotrienol, tocopherol, phytosterols, and other compounds in rice bran oil that reduced the generation of hydroperoxides. Also, as rice bran oil is richer in saturated FAs and MUFAs than SBO, the profile of the FA composition of the later could be modified, thus improving its thermal stability.
- *Genetic modification*. Genetic engineering technique goals include improving the oxidative and thermal stability of vegetal oils by altering the genetic properties of plants in order to increase the MUFAs content of the corresponding oil. A notable example by Burh et al.^86^ is the development of soybean seeds with oleic acid contents greater than 85% of the total oil by down-regulating the expression of FAD2 genes along with genes that control the production of palmitic acid. Currently, Plenish high-oleic SBO is commercially available for biodegradable lubricant formulations produced by DuPont Pioneer.^87^ High oleic soybeans are the only soybeans with genetically modified oil compositions that are now commercially used for industrial applications. More recently, Tsakraklides et al.^88^ reported the genetic engineering of a strain of the yeast species *Yarrowia lipolytica* that produced oil highly enriched in MUFAs and devoid of PUFAs (oleate content > 90% of total FAs) with properties as good or better than petroleum-based oils.
- *Additivation.* Apart from the natural antioxidants, recently, nanoparticles with antioxidant activity have been used. In recent papers by Tan and co-workers,^89,90^ hydrophilic zeolite nanoparticles containing extra-framework Ca^2+^ ions have been used as effective antioxidant additives to enhance oxidative stability of palm oil-based lubricants. The antioxidant activity of these nanoparticles was ascribed to three effects: (a) selective adsorption of hydroperoxides, (b) stabilization of a thermodynamically unstable O–O–H group of hydroperoxides, and (c) reduction of oil acidity by neutralizing the acidic carboxylate compounds to COO^–^(Ca^2+^)_1/2_ salts. As a result of these processes, decomposition of hydroperoxides is delayed and, consequently, C=C cleavage and propagation steps are decelerated, while oil acidity is decreased. Zaarour et al.^91^ demonstrated that Linde Type L zeolite (LTL) nanocrystals (15–20 nm) prevented the depletion of ZDDP (zinc dialkydithiophosphate, antiwear and antioxidant additive) at elevated temperature, thus extending its active life. At the same time, LTL adsorbed the secondary oxidation products generated, delaying the degradation of the lubricant. Ca^2+^-LTL and K^+^-LTL zeolite nanoparticles were found to be promisingly eco-friendly antioxidants due to their capability to hinder palm oil oxidation. Recently, carbon dots (CDs) obtained from tea wastes and glutathione/citric acid exhibited good solubility in nonaqueous media and could be used as green antioxidant additives in an ISO 68 base lubricant oil.^92^ The use of antioxidant nanoparticles as green additives in lubricants is scarce, and many studies still remain, particularly those related with the cytotoxicity of the CDs or the synthesis procedures that focus on green chemistry.
- *Chemical modifications*. This is an advantageous way to both mitigate some of the limitations and improve some tribological characteristics of vegetal-based lubricants. The modifications may be carried out in two different ways: (a) reactions at the double bonds of the FA chain (selective hydrogenation, epoxidation, dimerization/oligomerization) and (b) reactions at the carboxyl groups of FAs/TAGs/esters (include esterification/transesterification and estolide formation). In the following, we describe the most important features of such chemical modifications.

### Epoxidation

Epoxides (also known as oxiranes) are cyclic ethers with a reactive three-membered ring. Epoxidation of vegetal oils (also FFAs and esters) is the reaction through which the double bond in the unsaturated fraction is converted into epoxide groups by using peracids as epoxidizing agents. Organic peracids are formed by reacting acetic or formic acid with hydrogen peroxide in the presence of a strong acid (H_2_SO_4_ or H_3_PO_4_) to render performic acid (HCOOOH) and peracetic acid (CH_3_COOOH), respectively.

**Figure 7:**
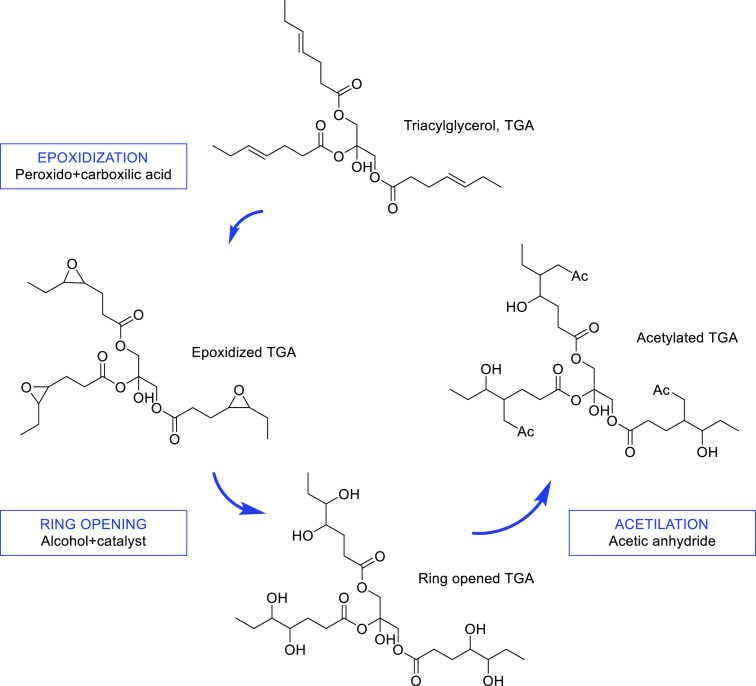
Epoxidation process of triacyglycerol (TAG) and formation of an acetylated product.

Epoxidation can be carried out either by homogeneous or heterogeneous catalysis. In homogeneous catalysis, the peracids are generated in situ by mixing the hydrogen peroxide with the acids. With the aim to avoid (a) the corrosive nature of this reaction media, (b) undesirable side reactions due to the epoxide ring opening, (c) cumbersome separation of acidic byproducts, (d) production of neutralized salts, and (e) low conversion, heterogeneous catalysis using ion exchange resins and transition metal-based catalysts (TiO_2_/SiO_2_, MoO_3_-Al_2_O_3_, MeReO_3_ on Nb_2_O_5_, etc.)^93−95^ are used for an environmental friendly and efficient process (Figure 7). Epoxidation can be also performed using enzymes such as *Candida antarctica* lipase B immobilized onto acrylic resins or silica. A review devoted to the chemoenzymatic epoxidation of FAs and vegetal oils has been recently published by Milchert et al.^96^

The epoxidized vegetal oils are more thermally stable than the parent TAGs and have superior oxidative stability due to the removal of the bis-allylic protons, allowing them to be used as high temperature lubricants. Wu et al.^97^ reported the epoxidation of rapeseed oil and its improved oxidation stability, with increased PP and lubricity as compared to neat oil. Epoxidized vegetal oils and epoxidized fatty esters/FAs can also be employed as antiwear/antifrictional additives for biodegradable vegetal oil lubes and synthetic esters, showing a better performance than conventional petroleum-based additives. Doll et al.^98^ examined the physical properties relevant to lubricant applications, oxidation onset temperature, PP, and VI of SBO-based olefins and epoxides (Table 1).

**Table 1:** Physical Properties of Soybean Oil-Based Olefins and Their Epoxides

Samples	Oxidation onset temperature (°C)	Pour point (°C)	Viscosity index (mm^2^ s^–1^)
**Neat olefins**			
SBO	155	–9	225
Methyl oleate	177	–27	199
Methyl linoleate	139	–48	Undefined
Methyl linolenate	117	–60	Undefined
**Epoxides**			
Epo-SBO	199	3	142
Epo-methyl oleate	190	0	151
Epo-methyl linoleate	180	–1.5	132
Epo-methyl linolenate	131	–7.5	63

Epoxidation of olefinic materials improved their oxidative stability and increased their adsorption to metal surfaces, compared to the corresponding neat olefins which, on the other hand, have better PPs and VIs. Naturally epoxidized TAGs can be found in natural oils from the two genuses Vernonia (*Vernonia galamensis*) and Euphorbia (*Euphorbia lagascae*), which contain up to 70%–80% of vernolic acid (12S,13R-epoxy-9-cis-octadecenoic acid).^99,100^ One species of Euphorbia (*Bernardia pulchella*) from Brazil has been determined to contain more than 90% vernolic acid in the TAGs.^101^ In spite of the high content of vernolic acid of these natural oil sources, their use as potential substitutes of chemically epoxidized oils is still in its infancy. Further epoxidation of these natural oils increases the epoxy units per TAG molecule. Desalegn Zeleke^102^ reported the epoxidation of vernonia oil with a 78% yield as a promising intermediate for synthesis of biolubricants. Some significant advances are being developed by genetically engineering the biosynthesis of epoxy acids in oil seeds. Cahoon et al.^103^ transferred into soybean seeds the capacity to produce high contents of vernolic acid. The expression of a cytochrome P450 epoxygenase from *Euphorbia lagascae* in somatic soybean resulted in an increase of vernolic acid up to 8% of the total FAs of the transgenic soybean embryos. Li et al.^104^ cloned the epoxygenase SlEPX responsible for vernolic acid synthesis from seeds of *S. laevis* and two acyl-CoA (VgDGAT1 and VgDGAT2) responsible for catalyzing TAG formation from *V. galamensis*, with the aim to develop transgenic soybeans able to co-express such enzymes. Co-expression of SlEPX and VgDGAT1 or VgDGAT2 increased accumulation of vernolic acid (up to a 25%) in soybean somatic embryos. Lubricant studies and/or applications of the oils from these epoxy-enriched seeds have yet to be performed. Oils from these soybean seeds with a high content of vernolic acid may open new opportunities for the “green” lubrication sector as they are expected to have improved stability and lubricity for metal surfaces. Looking forward to the future, it is not clear if these oils could be developed for commercial use, not only due to the elevated costs linked to the regulatory evaluation delays of genetically modified crops but also because of the secondary environmental concerns around its introduction.^105^

**Figure 8:**
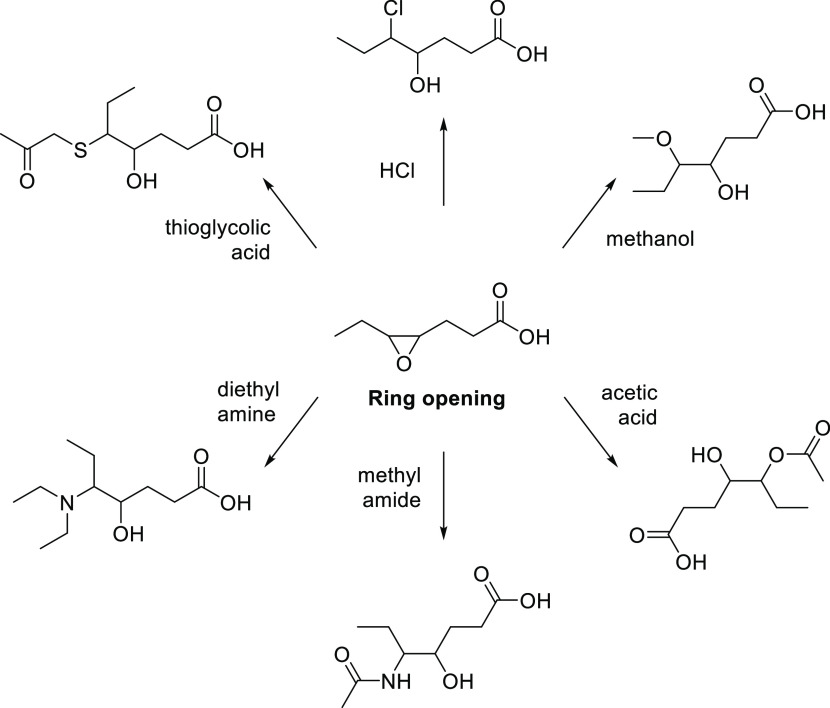
Examples of oxirane ring opening for the introduction of different functionalities to produce biodegradable lubes.

The epoxidation of vegetal oils/esters/FAs is often the previous step to ring opening that results in the production of different compounds with applications in the formulation of biodegradable lubricants. Ring opening proceeds through the cleavage of one of the C–O bonds, initiated by either electrophiles or nucleophiles. Ring opening can proceed by either S_N_2 or S_N_1 mechanisms, depending on the nature of the epoxide and on the reaction conditions. Oxirane ring opening allows the introduction of heteroatoms and many different functional groups that can be used as base lubricants and additives. In Figure 8, different functionalities introduced by ring opening reactions are depicted.

In 2013, Sharma and Dalai^106^ reported the simultaneous ring opening and esterification of epoxy canola oil in the presence of acetic anhydride and sulfated Ti-SBA-15 as the catalyst. Results demonstrated a 100% epoxy canola oil transformation to the esterified product, which in turn exhibited good tribological properties, such as an oxidative induction time of 56.1 h, a cloud point of −3 °C, a PP of −9 °C, and a kinematic viscosity at 100 °C of 670 cSt. Also, the biolubricant demonstrated excellent lubricity properties by a wear scar of 130 μm. Kulkarni et al.^107^ reported the epoxidation of mustard oil by using performic acid and nanoalumina as a catalyst and the subsequent acid-catalyzed ring opening with 2-ethylhexanol (2-EH). The 2-EH esters of epoxidized mustard oil have pour points of −35 °C and an enhanced VI. Harry-O’kuro et al.^108^ explored the syntheses of formyl esters of three epoxidized vegetal oils in order to examine those physical characteristics related with their suitability as lubricant candidates. The epoxy ring opening process for formyl ester generation was mild and was followed by a simultaneous condensation reaction of the putative α-hydroxy formyl intermediate to yield vicinal diformyl esters from the epoxy group. Results demonstrated that three polyformyl esters from milkweed, soy, and pennycress exhibited low CoF and a correspondingly low wear scar. More recently, Borugadda and Goud^109^ reported the epoxidation of waste cooking oil and waste cooling oil FA-methyl ester and the epoxides hydroxylated (ring opening) using various alcohols using sulfuric acid (homogeneous catalysis) and the ion-exchange resin IR-120 (heterogeneous catalysis). Tribologically significant properties such as PP, thermo-oxidative stability, rheology, and biodegradability were improved.

### Selective Hydrogenation

Hydrogen atoms in double bonds are bis-allylic protons and thus highly reactive. Elimination of such double bonds by selective/partial hydrogenation a fraction of PUFAs can be transformed into MUFAs, thus improving the oxidative stability and low-temperature properties of the oils. The hydrogenation of fats and oils is a complex process, as along with the addition of hydrogen to double bonds, dehydrogenation processes may occur. Consequently, stereoisomeric acids may be formed. It is not an easy task to control the entire reaction uniformly and selectively to avoid formation of saturates or trans products with high PPs, even by using different catalysts or by varying the reaction conditions. In fact, catalytic partial hydrogenation of TAGs always generates some trans isomers. The key for “good” selective and partial hydrogenation (unsaturation degree reduced as much as possible while limiting cis–trans and functional isomerization) is the adequate selection of the catalyst.

Traditionally, the hydrogenation process proceeds by flushing hydrogen gas into a reactor containing vegetal oil at high pressure (70–420 kPa) and temperatures ranging from 150 to 225 °C using a nickel catalyst on a silicate support. A natural silicate-diatomite nickel catalyst has been shown to be selective for monounsaturated oleic acids during SBO and sunflower oil hydrogenation in an industrial reactor at high temperature (165–200 °C) and hydrogen pressure (0.05–0.2 MPa).^110^ However, copper catalysts have been shown to have higher selectivity for hydrogenating linoleic acid during SBO hydrogenation, and this fact has been used to increase the SBO stability by selective hydrogenation of linolenic acid. Trasarti et al.^111^ reported the liquid-phase hydrogenation of SBO using copper catalysts, which exhibited unique properties for obtaining proper lubricants due to the selective hydrogenation of unsaturated linolenic (C18:3) and linoleic (C18:2) FAs to unsaturated oleic acid (C18:1), while saturated stearic acid (C18:0) was not formed. Although the advantages provided by Ni and Cu catalysts, such as low cost, easy removal from oils by filtration, and selectivity and some drawbacks such as the isomerization of natural cis to trans bonds during Ni-catalyzed hydrogenation and low activity at temperatures below 120 °C, noble metal catalysts (e.g., Pd, Pt, and Ru) are usually employed due to their high activity in small amounts at low temperature and the possibility of reuse.^112−114^ Each noble metal catalyst exhibits particular characteristics in selectivity, reactivity, and trans isomerization during hydrogenation of vegetal oils. It has been accepted that Pt catalysts produce the least amount of trans FA during hydrogenation, less than 8%, while conventional hydrogenation produces hydrogenated oils containing from 25 to 45% trans FAs. Also, the catalyst amount when using noble metal catalysts is about 1/10 to 1/40 of Ni catalysts in conventional hydrogenation. In contrast, the catalysis time is longer using noble metal catalysts due to the low reaction temperature and the low amount of catalyst employed.^115^ At the present time, no data have been documented in the oil industry for some drawbacks of using noble metal catalysts (particularly, Pt) such as the need to recover the catalytic activity of the material, loss of part of the material during the process, or reuse of the catalyst.

### Dimerization/Oligomerization

Dimerization/oligomerization methods are another technologically viable options to eliminate the double bonds of unsaturated FAs and FA esters. In general, PUFAs can be easily dimerized by heat treatment, while a catalyst is needed for dimerization of MUFAs. In the case of thermal dimerization at temperatures about 300 °C, side reactions of FFAs such as decarboxylation and anhydride formation result in low yield. For this reason, thermal dimerization is better performed using methyl-ester and TAGs than FFAs.^116^ Traditionally, oligomerization of FAs has been performed by two catalytic approaches: (a) homogeneous catalysis using alkali or alkaline metal salts, iodine, Lewis acids (e.g., SnCl_4_), and Brønsted acids (e.g., resin in H^+^ form) as catalysts and (b) heterogeneous catalysis, more environmentally friendly, using materials such as kaolinite, bentonite, and montmorillonite.^117,118^ Dimer acids can be synthesized by two identical or different unsaturated C_18_ FAs such as oleic acid, linoleic acid, tall oil, and other unsaturated FAs by reaction at about 210–250 °C in the presence of montmorillonite as the catalyst resulting in a mixture of cyclic and linear C_36_-dicarboxylic acids and C_54_-trimer FAs, as well as C_18_-monomeric FAs (mixture of saturated, unsaturated, straight chain, and branched). It is assumed that the mechanism through which the reaction proceeds over clays is a combination of Diels–Alder addition (one FA acts as the diene and the other as the dienophile), isomerization, conjugation, and hydration/dehydration reactions. Diesters based on short-chain linear diacids (C_6_–C_12_) exhibit low viscosity, high polarity, high VIs, and low PPs while, in comparison, esters obtained from C_36_ dimer acids have higher viscosity and lower polarity, and due to residual unsaturation and branching, thermal and oxidative stability are lower. However, Armylisas et al.^119^ reported the synthesis of four short-chain dimerate esters (dibutyl, dihexyl, di(2-ethylhexyl), and dioctyl dimerate) and their evaluation as lubricant base stocks. Results demonstrated that the materials exhibited high VIs and significantly low PPs, less than −42 °C for the di(2-ethylhexyl) dimerate, which was ascribed to the branching of the side chain. Also, the esters showed oxidative stability, attributed to the hydrogenation of residual double bonds.

### Esterification/Transesterification

Alcoholysis of TAGs is used to prepare alkyl esters, the most used method to modify the carboxyl group of the FAs. Esterification involves FFAs of natural oils reacting with long-chain alcohols to form the corresponding esters. During transesterification, glycerol moieties of the TAGs are replaced by long- and/or branched-chain alcohols. The transesterification process for green lubricant synthesis can be chemically or enzymatically catalyzed. A good example of these reactions include the transesterification of palm oil. The reaction proceeds in two-steps:^120^ (a) The FFAs and the TAGs are reacted with methanol in the presence of a basic catalyst to produce palm oil methyl esters. (b) The resulting palm oil methyl esters are reacted in the presence of a catalyst such as sodium methoxide (minimizes saponification of esters) with a polyhydric alcohol (e.g., trimethylolpropane, TMP) to produce the corresponding polyol esters and methanol. During the transesterification process, the ester group of palm methyl ester is replaced by the hydroxy group of TMP. An advantage of using polyhydric alcohols is the absence of β-hydrogen, which results in the enhancement of thermal and oxidative stability of the lubricant at high temperatures.^121^ Afifah et al.^122^ described the development of a green lubricant from palm stearin, a byproduct of palm oil, by enzymatic transesterification using *Candida antartica* lipase B as the catalyst and methanol in a solvent-free system at a maximum yield around 95%. The chemical modification of palm stearin resulted in improvement in both physicochemical and tribological properties, such as superior VI (>120) and friction properties over commercial mineral oil-based lubricants.

In general, the polyol esters formed by transesterification of vegetal oils show good biodegradability, possess high lubricity, provide corrosion protection, and have good oxidative stability, high VI, and good shear stability. The transesterification process results in a reduction of the intramolecular forces among the TAGs and FAs, thus reducing the viscosity of the product. Also, with removal of the polyunsaturation, the increase in the chain length, and branching of alkyl chains as a result of transesterification, improved PPs are observed when compared with the unmodified oil, thus meliorating its low-temperature performance.

The problem of oxidative stability, among the FAs esters, is minimal when saturated low molecular weight FAs are involved. Wang et al.^123^ demonstrated that TMP triesters of C_6_, C_8_, and C_10_ FAs showed reduced viscosity (23.3 mm^2^ s^–1^), a quite low PP (−45 °C), and high flash point (248 °C), along with a remarkable oxidation stability demonstrated by an oxidation induction time of 38 h at 130 °C, attributed to the elimination of C=C double bonds and β-H atoms. The use of saturated FAs of higher molecular weight is not practical due to the high melting point of esters. From an environmental point of view, the results presented by Makarevicine and Janulis^124^ are interesting; rapeseed oil ethyl esters were more rapidly biodegradable in a water environment than rapeseed oil methyl esters, highlighting the importance of using ethyl esters over the methyl ones.

### Estolide Formation

**Figure 9:**
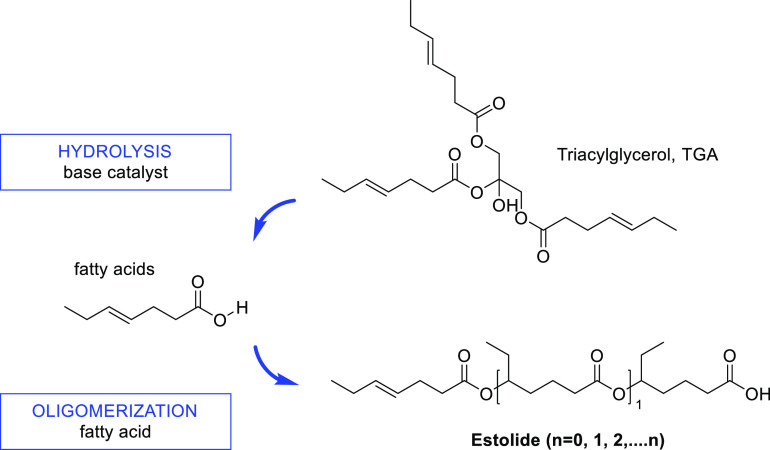
Process of formation of an estolide.

An estolide is formed through the condensation of the carboxylic acid group of one FA and the unsaturation of another FA, thus forming oligomeric esters (Figure 9). Estolides can be also formed by addition of a FA to a hydroxy containing FA. The extent of oligomerization (average number of FAs added to the base FA) is represented by the estolide number (EN).

The conventional chemical synthesis of estolides requires high temperatures (200–210 °C) or strong acids as catalysts which results in a low selectivity, coloring, bad odor, and unwanted byproducts that may cause equipment corrosion and acid effluents. Consequently, mild enzymatic synthesis has been described as a plausible alternative, and some enzymes such as *Avena sativa* L., *Rizopus oryzae* lipase, *Pseudomonas aeruginosa* KKA-5 lipase, or *Thermomyces lanuginose,* among others, have been used.^125,126^

Natural estolides have been reported to occur in plants that produce hydroxy FAs such as ricinoleoyl estolides from castor oil or TAG estolides in several plant genus like *Physaria*, *Heliophila amplexicaulis*, *Lesquerella*, *Nerium*, *Sapium sebiferum*, *Trewia nudiflora*, and *Avena sativa*. The best known of the hydroxy acids of the seeds of some of these plants are those found in lesquerella oil in which 55%–60% corresponds to the hydroxy FA lesquerolic (14-hydroxy-cis-11-eicosenoic acid) and 2%–4% to the auricolic acid (14-hydroxy-cis-11-cis-17-eicosenoic acid) and the ricinoleic acid D-(−)12-hydroxy-octadec-cis-9-enoic acid) which comprises up to 90% of castor oil (from *Ricinus communis*).^127^ The content of estolides in these natural sources is low and from a commercial point of view not attractive; however, synthetic estolides that mimic the natural ones can be commonly produced from the hydroxy moiety of lesquerella or castor oil. The properties of the synthetic estolides can vary widely by choice of capping FAs and the degree of estolide formation (capping) from single capped molecules to fully capped oils containing up to six ester linkages.

In general, the estolides exhibit improved lubricity, high VI, and low PP. The ester linkage of estolides is more resistant to hydrolysis than that of TAGs, thus having higher hydrolytic stability and exhibiting improved physical properties to be used as biolubricants. Although the oxidative stabilities of estolides are rarely informed, in a recent paper by Hoong et al.,^128^ it was reported that lauric acid capped estolide from oleic acid and branched with secondary amines to obtain estolide amides exhibited a high oxidative stability with an oxidation onset temperature of 205 °C, significantly higher than that of vegetal oil-based lubricants.

## Conclusions

With the increasing global industrialization, the lubricant market in combination with consumer demand for high quality eco-friendly lubricants have driven the development of new technologies such as the production of biodegradable lubricants from natural resources such as vegetal oils. Vegetal oils are effective as boundary lubricants due to their fatty acids composition that allows strong interactions with the lubricated surfaces, but also, they are easily (bio)degradable, with this property being both an advantage and a drawback. Vegetal lubricant oils are subject to oxidation more easily than mineral or synthetic lubricants. Remarkable advances have been made in organic synthesis, catalysis, and biotechnology to ameliorate the oxidative and thermal stability of vegetable oils, including, for example, transesterification, epoxidation, or estolide formation. Although the stabilization or modification of vegetal oils can make them a real alternative to replace, total or in part, mineral lubricants, thus being a response to the current need for biodegradable and easily disposable lubricants, most studies are still produced on a laboratory scale. The use of biolubricants is still small, and only a few have the “eco-label”. To advance the research for new eco-friendly lubricants, further studies are needed in the following areas: (a) evaluation of inexpensive and environmental friendly oil extraction processes in order to scale-up at the industrial level (use–reuse cycle), (b) development of inexpensive and environmentally friendly additives (e.g., use of vegetable wastes to extract antioxidants or to produce functional nanoparticles) that improve both the chemical and tribological properties, (c) deep characterization of the chemical stability of eco-friendly additives, such as natural antioxidants, under working conditions, and (d) improvement of the chemical processes and uses of nonedible vegetal oils as suitable feedstock to produce renewable biolubricants at prices competitive with those of synthetic and mineral oils.
